# The impact of early life exposure to *Plasmodium falciparum* on the development of naturally acquired immunity to malaria in young Malawian children

**DOI:** 10.1186/s12936-019-2647-8

**Published:** 2019-01-18

**Authors:** Priyanka Barua, James G. Beeson, Kenneth Maleta, Per Ashorn, Stephen J. Rogerson

**Affiliations:** 1 The Department of Medicine (RMH), Peter Doherty Institute for Infection and Immunity, The University of Melbourne, Melbourne, VIC 3000 Australia; 2 Burnet Institute, Melbourne, VIC 3004 Australia; 3 Department of Microbiology and Central Clinical School, Monash University, Melbourne, VIC 3800 Australia; 4 School of Public Health and Family Medicine, University of Malawi, Blantyre 3, Malawi; 5 Faculty of Medicine and Life Sciences, University of Tampere and Tampere University Hospital, 33100 Tampere, Finland; 6 Present Address: Department of Zoology, University of Dhaka, Dhaka, 1000 Bangladesh; 7 Present Address: Research and Development, Maternal, Newborn and Adolescent Health, World Health Organization, Geneva 27, 1211 Switzerland

**Keywords:** Episodes, Nutrient supplements, Randomized controlled trial, Merozoite antigens, Variant surface antigens, Seroprevalence

## Abstract

**Background:**

Antibodies targeting malaria blood-stage antigens are important targets of naturally acquired immunity, and may act as valuable biomarkers of malaria exposure.

**Methods:**

Six-hundred and one young Malawian children from a randomized trial of prenatal nutrient supplementation with iron and folic acid or pre- and postnatal multiple micronutrients or lipid-based nutrient supplements were followed up weekly at home and febrile episodes were investigated for malaria from birth to 18 months of age. Antibodies were measured for 601 children against merozoite surface proteins (MSP1 19kD, MSP2), erythrocyte binding antigen 175 (EBA175), reticulocyte binding protein homologue 2 (Rh2A9), schizont extract and variant surface antigens expressed by *Plasmodium falciparum*-infected erythrocytes (IE) at 18 months of age. The antibody measurement data was related to concurrent malaria infection and to documented episodes of clinical malaria.

**Results:**

At 18 months of age, antibodies were significantly higher among parasitaemic than aparasitaemic children. Antibody levels against MSP1 19kD, MSP2, schizont extract, and IE variant surface antigens were significantly higher in children who had documented episodes of malaria than in children who did not. Antibody levels did not differ between children with single or multiple malaria episodes before 18 months, nor between children who had malaria before 6 months of age or between 6 and 18 months.

**Conclusions:**

Antibodies to merozoite and IE surface antigens increased following infection in early childhood, but neither age at first infection nor number of malaria episodes substantially affected antibody acquisition. These findings have implications for malaria surveillance during early childhood in the context of elimination.

*Trials registration* Clinical Trials Registration: NCT01239693 (Date of registration: 11-10-2010). URL: http://www.ilins.org

## Background

Malaria is one of the leading causes of childhood morbidity and mortality, and *Plasmodium falciparum* infection is responsible for nearly all malaria deaths. The World Health Organization estimated that in 2017 there were 435,000 deaths due to malaria of which around 61% were in children below 5 years of age [1]. Children in malaria-endemic areas become most vulnerable to malarial infection at 4 to 6 months of age as maternally transferred antibodies wane [2] and they then begin to acquire their own antibodies in response to repeated infection. Naturally acquired immunity to malaria develops over time with continuous exposure to infection [3].

Antibodies play a crucial role in mediating acquired immunity to malaria. Blood stage merozoite antigens and variant surface antigens (VSA) expressed on infected erythrocytes (IE) are important targets of this protective immunity. Antibodies to merozoite antigens inhibit invasion of red blood cells (RBCs), prevent intra-erythrocytic growth [4], and promote opsonization for phagocytic clearance [5] and complement fixation [6]. Antibodies to merozoite antigens of sufficient magnitude and function appear to contribute to immunity [7]. In young children or those with limited malaria exposure, they may instead act as biomarkers of malaria exposure [8, 9], with potential to inform surveillance and control activities [10]. Among the tested antigens, merozoite surface protein 1 (MSP1) is the most copious protein found on the surface of the merozoite [11]. Crucial for the primary interaction between merozoites and RBCs in parasite invasion [12], MSP1 is a major target of opsonizing following natural exposure [13]. MSP2 is another abundant, GPI-anchored surface protein necessary for merozoite invasion. Increased IgG level against MSP2 was associated with increasing age, higher haemoglobin level and reduced parasitaemia suggesting its protective effect [14]. Erythrocyte binding antigen 175 (EBA175) is released from micronemes [15] and aggregates at the apical region of the merozoite surface. Antibodies to the RIII-V region of EBA175 have also been associated with protection from malaria [16–18]. Rhoptry-derived Rh2A9 help binding to the RBC receptors after the primary interaction between the RBC and merozoite surface proteins is completed. The level of IgG against Rh2A9 in children (5–14 years) was associated with lower risk of malaria [19].

Antibodies to VSA diminish malaria risk by obstructing cytoadhesion to different host receptors [20, 21] and initiating phagocytic clearance of IE [22]. Several studies have reported associations between levels of anti-VSA antibodies and protection against symptomatic malaria [23–26] but few studies have examined the dynamics of naturally occurring anti-VSA IgG in infants in a malaria-endemic setting [27, 28]. In one study [27], children up to 24 months of age did not acquire antibodies to VSA but in a high-transmission area of Tanzania [29], children had dramatic increases in antibodies to VSA from 1 to 2 years of age. Recent studies in Papua New Guinea suggest that acquired antibodies to VSA play an earlier role in immunity to malaria than antibodies to merozoite antigens [30]. These contradictory findings indicate the need for further studies to investigate the dynamics of naturally acquired immunity targeting both merozoite antigens and VSA in very young children.

This study examines the dynamics of antibody acquisition to multiple merozoite antigens, schizont extract and VSA in young children in response to ongoing exposure to malaria. The study was part of the International Lipid-based Nutrient Supplement (iLiNS) Project DYAD-Malawi randomized controlled trial (clinicaltrials.gov registration number NCT01239693). The original study reported that nutrient supplementation did not have any significant impact on anthropometric indices in 18 months old children [31]. Another sub-study from the same cohort [32] reported that malaria antibody acquisition (against the same antigens reported here) in early infancy was not improved by additional lipid-based nutrient supplementation. For this report, malaria antibody measurement at 18 months of age was related to concurrent malaria infection and to documented episodes of clinical malaria during early childhood to understand the impact of exposure on antibody acquisition.

## Methods

### Study location and participants

The study participants were 601 infants of 18 months of age from rural Malawi, participants in the iLiNS Project DYAD-Malawi trial. Detailed description of the trial design and supplements has been published elsewhere [33]. Briefly, pregnant women were randomly allocated to supplementation groups that received either iron and folic acid (IFA), multiple micronutrients (MMN) or 20 g of lipid based nutrient supplements (LNS) daily. After delivery, women in the IFA group received placebo, whereas MMN and LNS supplementation was sustained for 6 months post-partum. From 6 to 18 months of age, children in the LNS group received 10 g LNS twice daily.

### Detection of malaria

Children were followed up weekly at home and febrile episodes were investigated for malaria. Clinical malaria was defined as fever with axillary temperature above 37.5 °C and parasitaemia was confirmed by microscopy or rapid diagnostic test (RDT). For microscopy, slides were examined under 100× magnification and parasites were counted against 200 leucocytes. The RDT was the Clearview^®^ Malaria Combo (British Biocell International Ltd, Dundee, UK) which detects the proteins *P. falciparum* lactate dehydrogenase and histidine-rich protein 2.

### Preparation of plasma samples

Blood from participants was collected at the 18-month study visit. Plasma was separated by centrifugation and stored at − 80 °C prior to shipping on dry ice to Australia. Samples were thawed, heat inactivated at 57 °C for 45 min, and were stored at − 80 °C until assayed.

### Maintenance of parasite culture

Three *P. falciparum* lines were cultured as previously described [34]. The E8B-ICAM line binds to ICAM-1 and CD36 [35], and expresses group B/C *var* genes [36]. Rosetting line R29 expresses *var* genes from group A [37]. A 3D7-derived line had a group A *var* gene as its dominant transcript, but its binding phenotype was not defined [30]. Cultures were synchronized by hypotonic lysis using 5% sorbitol, and by regular gelatin flotation [38]. To select R29 for high levels of rosetting, it was subject to two rounds of gelatin flotation. After the first round, the pellet was collected and resuspended in gelatin with heparin lithium salt, (0.05 mg/ml Sigma Aldrich), added to disrupt rosettes, and the supernatant was collected.

### Measurement of IgG to merozoite antigens and schizont extract

Merozoite antigens MSP119kD, MSP2 (FC27 clone), EBA 175, and *P. falciparum* reticulocyte binding protein homologue 2 (Rh2A9) were expressed and purified as previously described [16, 19, 32, 39, 40], and schizont extract was prepared as previously described [41]. ELISAs were performed as previously described [32].

### Measurement of IgG levels against VSA

IgG antibody levels against VSA were measured by flow cytometry as previously described [32], and flow cytometry data were analysed as described [42].

### Data analysis

Data were analysed using Stata version 13.0 (StataCorp, Texas, USA) and graphed using GraphPad Prism version 5 (La Jolla, CA, USA). Antibody levels were measured as optical density (OD) for schizont and merozoite antigens, or as geometric mean fluorescence intensity (MFI) for VSA. They were expressed relative to the positive control which was a pooled plasma sample from malaria exposed individuals from Africa.

Seroprevalence was specified as the percentage of children whose relative antibody level was greater than the mean plus three standard deviations of the negative controls’ antibody levels. Negative controls were a panel of malaria-naïve Melbourne blood donors. Socio-economic status (SES) was derived from an inventory of key household assets (HHA) adapted from [43]. Chi squared tests were performed to test the differences in seropositivity. Mann–Whitney tests were performed for comparing antibody levels between two groups and Kruskal–Wallis tests were performed to compare the antibody levels among more than two groups.

The association between malaria episodes and antibody seroprevalence in 18 months old children was investigated using logistic regression, and linear regression was performed to study the association of malaria episodes with antibody levels. Linear regression was done by transforming antibody levels to their natural logarithm; these were back transformed for reporting descriptive results. Multivariate regression adjusted for the covariates duration of gestation, HIV infection, gender of the child and maternal anaemia.

## Results

### Study population characteristics

A total of 601 samples from 18 months old children were tested (48.8% male and 51.2% female). The nutrient supplements received by their mothers were IFA in 33.6%, MMN in 33.4%, and 32.9% received LNS. The mean haemoglobin level was 10.8 ± 1.5 g/dl with a prevalence of anaemia (haemoglobin level ≤ 10.9 g/dl) of 46.3%. The percentage of children having *P. falciparum* parasitaemia at the 18-month visit was 6.5% by microscopy and 9.9% by RDT (Table 1).

**Table 1:** Study population characteristics

Characteristics of the participants	Number (%) [n = 601]
Female	308 (51.2)
Blood haemoglobin concentration, mean ± SD, g/dl	10.8 ± 1.5
Anaemia^a^	278 (46.3)
Parasitaemia by microscopy	39 (6.5)
Parasitaemia by RDT^b^	60 (9.9)
Number of children with malarial episodes	144 (23.9)
Single episode	119 (19.8)
Multiple episodes	25 (4.2)
Low socio-economic status	357 (59.4)
Mother’s education below median	321 (53.4)

^a^Anaemia defined as Hb ≤ 10.9 g/dl

^b^Rapid diagnostic test

### Magnitude and prevalence of antibodies and IgG responses in parasitaemic and aparasitaemic children at 18 months

Antibody seroprevalence against merozoite antigens, schizont extract and VSA was measured for all the tested children (Table 2). The seropositivity was highest for MSP1 (54.4%), followed by schizont extract (54.1%). However, IgG against VSA for all the tested parasite lines were very low and few children were seropositive.

**Table 2:** Magnitude and prevalence of antibodies and IgG responses in parasitaemic and aparasitaemic children at 18 months

Antigen tested/parasite isolate	Median antibody level^a^ (IQR^b^) Number of seropositive^c^ children (%)			p value^d^
	All children; (n = 601)	Parasitaemic children; (n = 60)	Aparasitaemic children; (n = 541)	
MSP1 19kD^e^	2.80 (0.65, 7.09)	12.31 (4.38, 28.79)	2.37 (0.56, 6.08)	*<* *0.0001*
	327 (54.4)	53 (88.3)	274 (50.7)	*< 0.0001*
MSP2^f^	2.91 (0.87, 9.04)	15.14 (3.47, 35.16)	2.62 (0.79, 7.28)	*< 0.0001*
	162 (26.9)	36 (60.0)	126 (23.3)	*< 0.0001*
EBA175^g^	3.38 (1.95, 16.09)	5.89 (2.89, 21.37)	3.24 (1.92, 15.89)	*0.0228*
	69 (11.5)	15 (25.0)	54 (9.9)	*0.001*
Rh2A9^h^	7.17 (2.1, 25.3)	8.59 (3.28, 19.24)	7.11 (4.05, 13.27)	0.5630
	151 (25.1)	23 (38.3)	128 (23.7)	*0.013*
Schizont	3.30 (1.27, 8.09)	17.31 (5.22, 48.66)	2.95 (1.09, 6.98)	*< 0.0001*
	325 (54.1)	58 (96.7)	267 (49.4)	*< 0.0001*
E8B^i^	0 (0, 0.20)	0.15 (0, 0.86)	0 (0, 0.16)	*< 0.0001*
	43 (7.2)	9 (15.0)	34 (6.3)	*0.013*
R29^i^	0 (0, 0.07)	0 (0, 1.12)	0 (0, 0.002)	*< 0.0001*
	21 (3.5)	9 (15.0)	12 (2.2)	*< 0.0001*
3D7^i^	0 (0, 0.35)	0.30 (0, 2.08)	0 (0, 0.29)	*< 0.0001*
	50 (8.3)	17 (28.3)	33 (6.1)	*< 0.0001*

Significant *p* value in italic format

^a^Antibody levels expressed as units relative to positive control

^b^Interquartile range

^c^Samples defined as antibody positive if levels greater than mean + 3SD of negative controls

^d^p value between parasitaemic and aparasitaemic children calculated by Mann–Whitney test or Chi squared test for antibody level or seropositivity, respectively

^e^Merozoite surface protein 1

^f^Merozoite surface protein 2

^g^Erythrocyte binding antigen 175

^h^Reticulocyte binding protein homologue 2A

^i^Variant surface antigen antibody responses

Children who were parasitaemic (n = 60) by RDT (9.9%) at the time of sample collection at 18 months had higher antibody levels than aparasitaemic children, and differences were statistically significant for all the tested antigens except Rh2A9. The seroprevalence data were also in accordance with the antibody level data and parasitaemic children were more frequently seropositive for all the antigens than the aparasitaemic children (Table 2). Children who were parasitaemic at 6 months (n = 57) were somewhat more likely to experience malarial episodes from 6 to 18 months of age; 14 out of 57 (24.6%) parasitaemic children at 6 months had episodes between 6 to 18 months compared to 99 out of 544 (18.2%) aparasitaemic children (p = 0.24, Chi square).

### Association of previous malaria episodes with antibody seroprevalence in 18 months old children

One-hundred and forty-four children (23.9%) had one or more malaria episodes before 18 months of age. Antibody seroprevalence was higher in the children with previous clinical malaria episodes than those without malaria for all the tested antigens, and differences were statistically significant for MSP1, MSP2 and schizont extract and IgG against E8B VSA (Table 3).

**Table 3:** Association between malaria episodes and antibody seroprevalence in 18 months old children

Antigen tested/parasite isolate	Seropositives^a^ (%)	No episode (n = 457)	Any episode within 18 months % (n = 144)	p value^b^	Single episode % (n = 119)	p value^b^	Multiple episodes % (n = 25)	p value^b^	Unadjusted OR (95% CI)	p value^c^	Adjusted OR (95% CI)	p value^d^
MSP1 19 kD^e^	327 (54.4)	224 (49.0)	103 (71.5)	*<* *0.0001*	83 (69.8)	*<* *0.0001*	20 (80.0)	*0.003*	2.24 (1.59, 3.17)	*<* *0.0001*	2.21 (1.56, 3.12)	*<* *0.0001*
MSP2^f^	162 (26.9)	113 (24.7)	49 (34.0)	*0.028*	40 (33.6)	0.051	9 (36.0)	0.207	1.41 (1.02, 1.94)	*0.034*	1.38 (1.00, 1.90)	*0.049*
EBA175^g^	69 (11.5)	50 (10.9)	19 (13.2)	0.459	13 (10.9)	0.996	6 (24.0)	*0.047*	1.33 (0.87, 2.05)	0.182	1.31 (0.85, 2.01)	0.216
Rh2A9^h^	151 (25.1)	108 (23.6)	43 (29.9)	0.133	34 (28.6)	0.265	9 (36.0)	0.160	1.32 (0.95, 1.83)	0.095	1.30 (0.93,1.80)	0.117
Schizont	325 (54.1)	222 (48.6)	103 (71.5)	*<* *0.0001*	85 (71.4)	*<* *0.0001*	18 (72.0)	*0.023*	2.14 (1.52, 3.00)	*<* *0.0001*	2.20 (1.55, 3.13)	*<* *0.0001*
E8B^i^	43 (7.2)	24 (5.3)	19 (13.2)	*0.001*	17 (14.3)	*0.001*	2 (8.0)	0.554	1.85 (1.15, 2.98)	*0.010*	1.87 (1.15, 3.04)	*0.011*
R29^i^	21 (3.5)	13 (2.8)	8 (5.6)	0.122	7 (5.9)	0.107	1 (4.0)	0.738	1.55 (0.78, 3.09)	0.204	1.51 (0.75, 3.02)	0.245
3D7^i^	50 (8.3)	36 (7.9)	14 (9.7)	0.485	10 (8.4)	0.850	4 (16.0)	0.152	1.31 (0.80, 2.14)	0.278	1.30 (0.79, 2.14)	0.291

Significant *p* value in italic format

^a^Samples defined as antibody positive if levels greater than mean + 3SD of negative controls

^b^p value compared to children with no episode calculated by Chi squared test

^c^p-value derived from logistic regression with Odds Ratios (OR) and 95% confidence intervals (CI)

^d^p-value derived from multivariate logistic regression with Adjusted Odds Ratios (OR) after controlling for duration of gestation, HIV infection, gender of the child, maternal anaemia

^e^Merozoite surface protein 1

^f^Merozoite surface protein 2

^g^Erythrocyte binding antigen 175

^h^Reticulocyte binding protein homologue 2A

^i^Variant surface antigen antibody responses

To determine whether the percentage of seropositivity at 18 months of age varies according to the number of malaria episodes, children were divided into groups having either a single episode of malaria or more than one episode of malaria (detected by RDT). One-hundred and nineteen children had single episodes of malaria whereas 25 children had multiple episodes recorded. Antibody seroprevalence was higher in children having a single episode or multiple episodes than those without any episode detected. For a single episode, differences in prevalence were statistically significant for MSP1, schizont extract (p ≤ 0.051) and IgG against VSA expressed by the E8B parasite line (p = 0.001). In the smaller number of children having multiple episodes, prevalence of antibody to MSP1 (0.003), EBA175 (0.047) and schizont extract (0.023) was significantly higher than in children without malaria. There were no significant differences in antibody seroprevalence between those with single or multiple episodes.

Logistic regression revealed that children with any episode of malaria from birth to 18 months of age had significantly higher odds of being seropositive for MSP1, MSP2, schizont extract and VSA against E8B parasite line in both unadjusted (p ≤ 0.034) and adjusted analysis (p ≤ 0.049) than children without any episode.

### Association of previous malaria episodes with levels of antibodies in 18 months old children

**Fig. 1:**
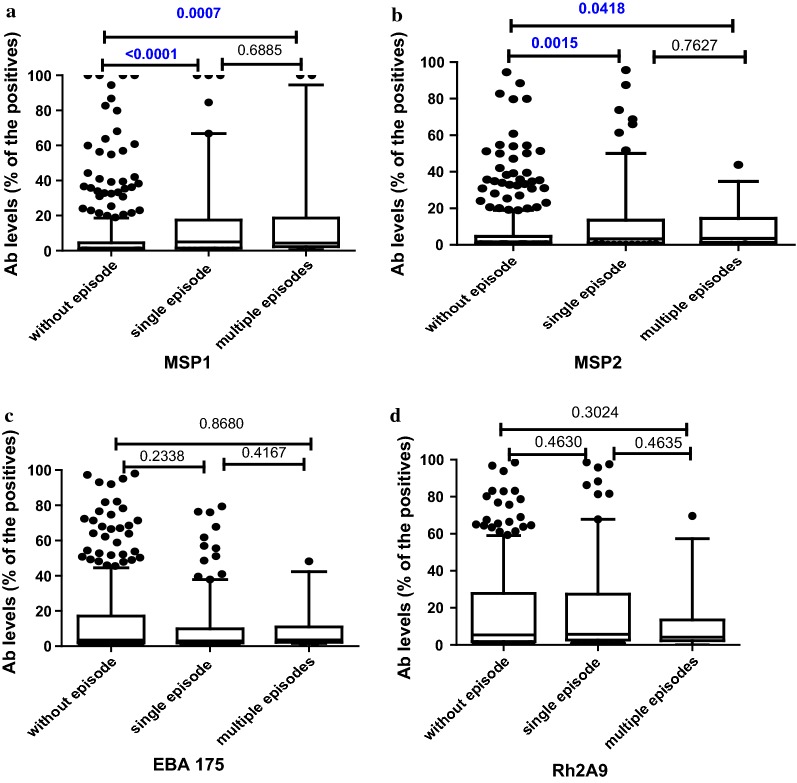
Levels of antibody at 18 months for **a** MSP1, **b** MSP2, **c** EBA175 and **d** Rh2A9, by history of episodes of malaria; (N; Without episode = 457, Single episode = 119, Multiple episodes = 25). Data presented as box plots with the Y axis representing antibody levels as a percentage of positive control (pooled plasma of malaria immune adults). The boxes denote the 25th to 75th percentile while the whiskers denote the 10th and the 90th percentile with outliers. p value calculated using Mann–Whitney test between the two groups indicated by the line

**Fig. 2:**
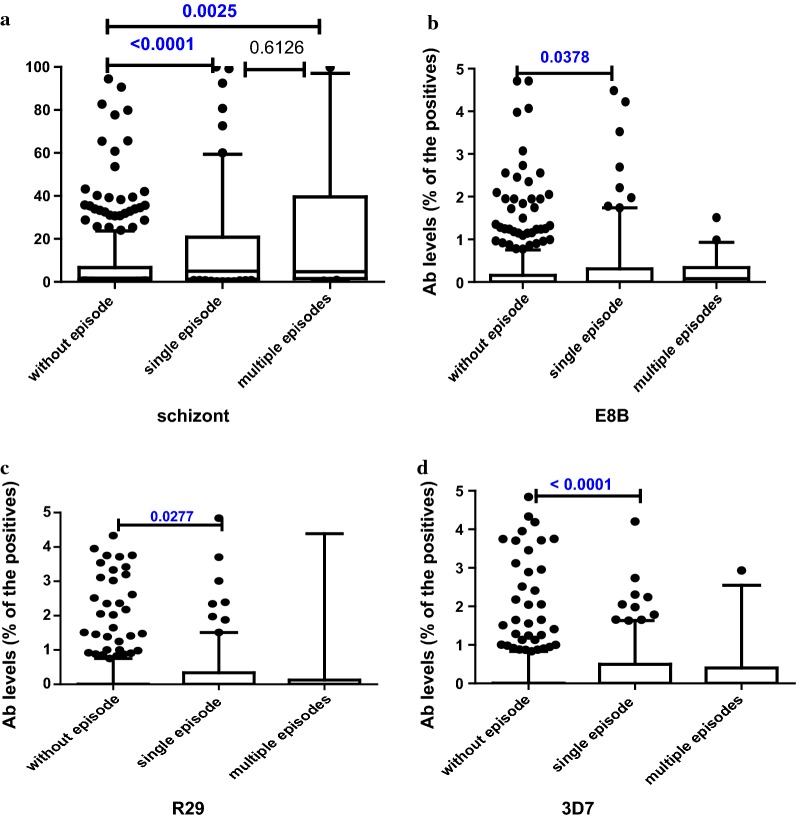
Levels of antibody at 18 months for **a** schizont extract, **b** E8B, **c** R29 and **d** 3D7 parasite line, by history of episodes of malaria; (N; Without episode = 457, Single episode = 119, Multiple episodes = 25). Data presented as box plots with the Y axis representing antibody levels as a percentage of positive control (pooled plasma of malaria immune adults). The boxes denote the 25th to 75th percentile while the whiskers denote the 10th and the 90th percentile with outliers. p value calculated using Mann–Whitney test between the two groups indicated by the line

The levels of antibody to malaria antigens were compared between infants who did and did not have previous malaria episodes (Figs. 1, 2). As observed with antibody seropositivity, children having malaria episodes had higher levels of antibodies to several antigens when compared to children without malaria history, and this was significant for MSP1, MSP2 and schizont extract. However, there were no significant differences between the antibody levels of children with single or multiple episodes for any antigen.

Levels of IgG against VSA were significantly higher for all the tested parasite lines in children having single malaria episode than those who had none (p value 0.038 for E8B, 0.028 for R29 and < 0.0001 for 3D7).

Linear regression (Table 4) indicated that having single or multiple episodes of malaria from birth to 18 months of age was associated with significantly higher antibody levels for MSP1, MSP2 and schizont extract in unadjusted analysis (p ≤ 0.047) and for MSP1 and schizont extract in adjusted analysis (p < 0.0001) for 18 months old children.

**Table 4:** Association between number of malaria episodes and antibody level in 18 months old children

Antigen tested/parasite isolate	Unadjusted coefficient (95% CI)	p value^a^	Adjusted coefficient (95% CI)	p value^b^
MSP1 19 kD^c^	1.85 (1.43, 2.41)	*<* *0.0001*	1.83 (1.41, 2.37)	*<* *0.0001*
MSP2^d^	1.28 (1.00, 1.63)	*0.047*	1.26 (0.99, 1.61)	0.057
EBA175^e^	0.92 (0.75, 1.11)	0.399	0.91 (0.76, 1.10)	0.378
Rh2A9^f^	1.14 (0.98, 1.33)	0.074	1.14 (0.98, 1.33)	0.082
Schizont	1.52 (1.21, 1.91)	*<* *0.0001*	1.50 (1.19, 1.87)	*<* *0.0001*
E8B^g^	1.06 (0.75, 1.50)	0.719	1.03 (0.73, 1.46)	0.826
R29^h^	1.33 (0.79, 2.24)	0.267	1.39 (0.81, 2.40)	0.222
3D7^h^	1.16 (0.79, 1.72)	0.434	1.13 (0.77, 1.67)	0.508

Significant *p* value in italic format

^a^p-value derived from linear regression of antibody levels and reported as coefficient and 95% confidence intervals (CI)

^b^p-value calculated using multivariate linear regression of antibody levels reporting coefficient and 95% confidence intervals (CI) while adjusting for duration of gestation, HIV infection, gender of the child, maternal anaemia

^c^Merozoite surface protein 1

^d^Merozoite surface protein 2

^e^Erythrocyte binding antigen 175

^f^Reticulocyte binding protein homologue 2A

^g^Variant surface antigen antibody responses

### Association of age at the time of malaria episode on antibody levels and seroprevalence in 18 months old children

To investigate whether age at the time of clinical malaria affected antibody levels or seroprevalence at 18 months, antibody levels and seroprevalence were compared between children who had malarial episodes prior to 6 months and those who had them between 6 and 18 months of age (Table 5). Both children who had malaria episodes before 6 months of age and those who had malaria episodes from 6 to 18 months had higher antibody levels and seropositivity to multiple antigens compared to those without any history of clinical malaria. However, antibody level or seroprevalence for all of the tested antigens did not differ between children who had malaria before 6 months or from 6 to 18 months.

**Table 5:** Association between age at the time of malaria episode and antibody levels and seroprevalence in 18 months old children

Antigen tested/parasite isolate	No episode^a^ (n = 457)	≥ 1 episode before 6 months of age^a^ (n = 31)	p value^b^	≥ 1 episode between 6 and 18 months of age^a^ (n = 113)	p value^b^
MSP1 19 kD^c^	2.36 (0.51, 5.73)	6.16 (1.92, 11.16)	*0.0071*	4.71 (1.43, 21.25)	*<* *0.0001*
	224 (49.0)	24 (77.5)	*0.002*	79 (69.9)	*<* *0.0001*
MSP2^d^	2.76 (0.79, 8.23)	5.76 (1.01, 10.55)	0.1043	3.06 (1.06, 13.97)	0.3165
	113 (24.7)	12 (38.8)	0.084	37 (32.7)	0.083
EBA175^e^	3.49 (1.96, 17.05)	3.25 (1.89, 8.79)	0.5785	3.19 (1.93, 9.91)	0.3753
	50 (10.9)	3 (9.7)	0.827	16 (14.2)	0.338
Rh2A9^f^	7.03 (3.97, 13.13)	8.24 (4.41, 17.32)	0.3790	7.92 (4.12, 15.87)	0.2223
	108 (23.6)	10 (32.3)	0.278	33 (29.2)	0.219
Schizont	2.95 (1.02, 7.24)	7.40 (2.24, 13.97)	*0.0211*	4.70 (1.77, 14.87)	*0.0018*
	222 (48.6)	23 (74.2)	*0.006*	80 (70.8)	*<* *0.0001*
E8B^g^	0 (0, 0.15)	0.03 (0, 1.11)	*0.0177*	0 (0, 0.26)	0.0967
	24 (5.3)	10 (32.3)	*<* *0.0001*	9 (7.9)	0.269
R29^g^	0 (0, 0.002)	0 (0, 0.43)	*0.0424*	0 (0, 0.24)	0.2341
	13 (2.8)	5 (16.2)	*<* *0.0001*	3 (2.6)	0.913
3D7^g^	0 (0, 0.33)	0.01 (0, 0.37)	0.4718	0 (0, 0.42)	0.8673
	36 (7.9)	4 (12.9)	0.324	10 (8.9)	0.734

Significant *p* value in italic format

^a^For each antigen represented, row 1: median antibody level (interquartile range); row 2: number of seropositive children (%)

^b^p value calculated by Mann–Whitney test or Chi squared test for antibody level or seropositivity, respectively

^c^Merozoite surface protein 1

^d^Merozoite surface protein 2

^e^Erythrocyte binding antigen 175

^f^Reticulocyte binding protein homologue 2A

^g^Variant surface antigen antibody responses

## Discussion

Naturally acquired immunity to malaria is achieved with ongoing exposure to infections and subsequent acquisition of anti-malarial antibodies. Antibodies against merozoite antigens and VSA are thought to play key roles in conferring immunity against malaria [26, 44]. The aim of this study was to examine how asymptomatic parasitaemia at the time of blood sampling and episodes of clinical malaria in young children affect the early development of antibodies against merozoite antigens and VSA. This study investigated whether the number of episodes in early life, or the age at which they occurred, influenced the development of antibody. The study found that at 18 months of age children who were parasitaemic had significantly higher levels of antibodies and seroprevalence to all the tested VSA and merozoite antigens than the aparasitaemic children, with the exception of antibody to Rh2A9. Children who had experienced clinical malaria episodes before 18 months of age had higher antibody levels and seroprevalence for the tested antigens than children who did not have any episode, and the levels and seroprevalence of antibodies at 18 months of age did not differ depending on the age of the child at the time of malaria episodes. This knowledge is relevant to informing vaccine development and strategies for sero-surveillance of malaria [10].

Previous studies have shown that newborn babies and young infants are relatively protected from symptomatic malaria [45, 46], and this has been attributed mainly to maternally transferred antibodies present in the first few months of life [2, 47]. Young children become most susceptible to infection as maternally derived antibodies wane, and then gradually begin to acquire antibody in response to infections, thus developing naturally acquired immunity to malaria [2, 3].

The present study examined the effect of clinical malaria episodes and parasitaemia on acquisition of antibodies by 18 months of age. At this time, little or no placentally transferred antibody remains, so antibodies elicited are likely to reflect naturally acquired immunity [47]. Antibody levels against all the tested antigens were relatively low, in agreement with earlier studies showing the age dependent acquisition of antibody [48, 49]. The proportion of children with detectable antibodies was highest for MSP1 (54.4%) and schizont extract (54.1%) perhaps because schizont extract acts as a crude marker of blood stage malaria infection whereas MSP1 is the most abundant merozoite surface protein [11]. Antibodies to VSA expressed by IE predominantly consist of antibodies to PfEMP1, the main antigen on the IE surface [50]. Antibodies to VSA are largely strain-specific and repeated exposure leads to the acquisition of a repertoire of antibodies to different variants [51]. Slow acquisition of variant-specific antibody, and possible lack of infection with variants similar to those tested, could explain the very low prevalence of detected antibodies to VSA.

Children who were parasitaemic at the time of sample collection at 18 months had significantly higher levels of antibodies and seroprevalence to all the tested VSA and merozoite antigens (except Rh2A9) than the aparasitaemic children. This finding is consistent with studies from Kenya in which antibodies to VSA [52] and to merozoite antigens [53] were higher in currently parasitaemic individuals. In the latter study, there were more dramatic differences in IgG levels between parasitaemic and aparasitaemic children, than between parasitaemic and aparasitaemic adults. Over two time periods of higher and lower transmission, parasitaemia was strongly associated with higher antibody levels for MSP2 and Rh2A9 (and to other antigens including AMA1 and MSP4), and weakly with antibody to MSP1, EBA175 and schizont extract [53]. In Malawi, by contrast, antibodies to all tested antigens except Rh2A9 were strongly associated with parasitaemia. However, it is possible that some infected children may have been missed because parasitaemia was detected using RDT, which may miss low density infections. Selecting antibody targets for surveillance of malaria exposure [10] will require evaluation in multiple populations and age groups.

The relationship between antibody levels or seroprevalence and history of clinical malaria infections was also investigated. Both measures were higher in children who had experienced clinical malaria episodes before 18 months of age than children who did not, and the differences were significant for MSP1 19 kd, MSP2, schizont extract, and (for antibody levels, but not seroprevalence) for IgG against all tested parasite lines. This is in accordance with other studies that report antibody boosting following exposure to clinical malaria [9, 27, 53–55], and suggests that merozoite (and VSA) antibodies act primarily as biomarkers of exposure in very young children [8, 9]. Some children with no malaria episodes detected did have antibodies; this likely reflects the occurrence of low-density asymptomatic infections that were not detected during follow up and were sufficient to generate antibodies to some antigens.

When the cohort was divided into children with single or multiple malaria episodes, those with single malaria episodes had significantly higher levels of antibody against MSP1, MSP2, schizont extract (≤ 0.0015) and IgG against VSA for all three tested parasite lines (≤ 0.028) compared to those without episodes. Fewer children had multiple episodes, limiting statistical power, but these children also had significantly higher antibody levels against MSP1, MSP2 and schizont extract (≤ 0.0418) than those who did not have malaria. There was no relationship between the number of clinical episodes and either antibody levels or prevalence. These findings suggested that antibodies to MSP1, MSP2 or schizont extract were useful markers of history of clinical malaria, but antibodies to EBA175 and Rh2A9 were not. In Kenyan children 1–8 years old, MSP1, MSP2, Rh2A9 and schizont extract also showed moderate induction following malaria episodes in the previous year, while EBA175 did not [53]. In sum, antibodies to MSP1, MSP2 and schizont protein extract may serve as good biomarkers for sero-surveillance of malaria [10, 56], although other antigens such as AMA1 and MSP4 [53] warrant further evaluation.

Infants’ immune systems are rapidly developing, and responses to infection may differ between younger and older children in magnitude or longevity. To investigate this, antibody levels and prevalence at 18 months were compared between children having malaria before 6 months of age, or from 6 to 18 months. Symptomatic malaria is uncommon in very young infants [3], and only 31 participants (5.15%) had clinical malaria before 6 months of age, whereas 113 (18.8%) had malaria from 6 to 18 months of age. The levels and seroprevalence of antibodies at 18 months of age did not differ between these two groups, and both children with malaria before 6 months of age and children with malaria from 6 to 18 months had higher levels of antibody (except to EBA175) than children with no malaria. This is in keeping with observations suggesting that the time of first exposure to malaria or previous malaria episodes have no effect on the acquisition of antibodies to MSP1, EBA175 and VSA [27, 28], although an earlier longitudinal study of antibody to MSP1 in infants showed highly dynamic antibody responses, with generally short-lived IgG peaks that correlated with symptomatic or asymptomatic infection [47]. Development and persistence of malaria antibody responses in infancy require further exploration.

The strengths of this study include the longitudinal design, with home-based monitoring of children with weekly home visits to 18 months of age and prompt detection and diagnosis of clinical malaria, and the number of antibody assays performed in a large group of over 600 well-characterized infants. The study provides unique insight into the development of antibody responses to VSA in relation to clinical malaria in infancy. Possible study weaknesses include the infrequent blood sampling of asymptomatic children, which gave only limited insights into the relationship between asymptomatic infection and antibody acquisition.

## Conclusions

The study provides a considerable insight on the acquisition of antibodies in early infancy in response to concurrent parasitaemia and clinical malaria. This study found that antibodies to tested merozoite and IE surface antigens increased following infection in early childhood and in response to concurrent parasitaemia at 18 months of age, but neither age at first infection nor number of malaria episodes substantially affected the antibody acquisition. The result provides strong evidence that antibodies to blood stage malaria antigens may be biomarkers of infection in early life and this knowledge is relevant to informing vaccine development and strategies for sero-surveillance of malaria.

## Abbreviations

EBA: erythrocyte binding antigen
HHA: household assets
IE: infected erythrocytes
IFA: iron and folic acid
iLiNS: International Lipid-based Nutrient Supplement
LNS: lipid-rich nutrient supplement
MMN: multiple micronutrients
MSP: merozoite surface protein
OR: odds ratio
PfEMP1: *P. falciparum* erythrocyte membrane protein-1
RDT: rapid diagnostic test
Rh2A9: reticulocyte binding protein homologue 2A
SES: socio-economic status
VSA: variant surface antigen
